# Editorial: Individual Differences in Arithmetical Development

**DOI:** 10.3389/fpsyg.2019.02672

**Published:** 2019-12-03

**Authors:** Ann Dowker, Bert De Smedt, Annemie Desoete

**Affiliations:** ^1^Experimental Psychology, University of Oxford, Oxford, United Kingdom; ^2^Faculty of Psychology and Educational Sciences, KU Leuven, Leuven, Belgium; ^3^Department of Experimental Clinical and Health Psychology, Ghent University, Ghent, Belgium

**Keywords:** mathematical cognition, individual differences, mathematical development, domain general abilities, numerical abilities, children, educational assessment, educational interventions

Individual differences in arithmetical performance have been known for a long time to be very marked in both children and adults (Dowker, 2005). For example (Cockcroft, 1982), reported that an average British class of 11-year-olds is likely to contain the equivalent of a 7-year range in arithmetical ability; and similar results were obtained 20 years and several educational changes later by Brown et al. (2002). Individual differences in arithmetic among children of the same age are also very great in most other countries. Such individual differences often appear to persist through life. At one end of the scale, about 22% of adults in the UK experience severe difficulties with basic numeracy, to an extent that leads to significant problems with employment and other everyday life activities. At the other end of the scale, some adults have an extreme fascination with numbers, can reason extremely well about numbers, and/or are exceptionally rapid and efficient calculators (Lubinski and Benbow, 2006).

There is increasing evidence that not only are there significant individual differences in children's arithmetic, but also that arithmetical ability is not unitary, but is made up of many different subcomponents (Jordan et al., 2009; Cowan et al., 2011; Desoete, 2015; Dowker, 2015; Pieters et al., 2015) and that individuals can show marked discrepancies, in both directions between different components: e.g., oral and written arithmetic; factual and procedural knowledge; exact calculation and estimation.

Individual differences in arithmetic are also increasingly studied from the point of view of their relation to more domain-general cognitive abilities, especially working memory and other executive functions. There is much evidence for significant relationships between executive functions and arithmetic (Bull and Scerif, 2001; De Smedt et al., 2009; De Weerdt et al., 2013; Bull and Lee, 2014; Peng et al., 2016; Bellon et al., 2019). Most studies have looked at executive functions as predictors of arithmetic; but there is some evidence for bidirectional relationships between the two (Welsh et al., 2010; Clements et al., 2016).

Individual differences in arithmetic include not only strictly cognitive factors but emotional ones as well. (Dehaene, 1997 p. 225) pointed out that, even when studying the neural aspects of mathematics, it is important to take emotional factors into account: “…cerebral function is not confined to the cold transformation of information according to logical rules. If we are to understand how mathematics can become the subject of so much passion or hatred, we have to grant as much attention to the computations of emotion as to the syntax of reason.” In particular, mathematics anxiety, sometimes amounting to real fear of mathematics is a very common phenomenon and is significantly negatively correlated with mathematical performance (Hembree, 1990; Ma and Kishor, 1997; Carey et al., 2016; Dowker et al., 2016; Foley et al., 2017; Sorvo et al., 2017; Zhang and Kong, 2019).

The study of individual differences in arithmetic, from all these perspectives has important implications for mathematics education and in particular for interventions with children with mathematical difficulties (Butterworth et al., 2011; Clements and Sarama, 2011; Chodura et al., 2015; Dowker, 2017).

The articles in this special issue are extremely diverse, reflecting a very varied area; but may be divided into the following broad categories: (1) the extent, nature and persistence of individual differences in mathematics, including methods of assessing these; (2) the componential nature of arithmetical ability, and discrepancies between different aspects of arithmetical cognition and performance; (3) the relationship between arithmetic and cognitive characteristics; (4) the relationships between mathematical performance and mathematics anxiety; and (5) implications of findings about individual differences for interventions for children with arithmetical difficulties.

(1) The nature and assessment of individual differences in arithmetic.

Mejias et al. studied the assessment of early mathematical abilities in school beginners. They developed a Mathematical School Readiness test assessing early mathematical abilities. In their study, 346 children, with a mean age of 6; 3 years, were given this test entering first grade, and it was found to correlate with classical curriculum mathematics test at the time, and also to predict later performance on such tests in second grade, thus suggesting that it may be a useful test for assessing school beginners' readiness for studying mathematics, and in particular for identifying children at risk for experiencing mathematical difficulties.

Greisen et al. investigated ways of assessing mathematics that do not depend on language. This is important for children who have language difficulties, or who are receiving their instruction in a language other than their native language; and also in comparing children from countries that speak different languages. The researchers developed video and animation-based task instructions on touchscreen devices that require no verbal explanation. These tasks were administered to two groups of children in the first grade of primary school in Luxembourg. One group (*n* = 96) received verbal instructions and the other group (*n* = 141) got video instructions. One group completed the tasks with verbal instructions while another group received video instructions. Overall, the groups performed similarly, indicating that explicit verbal instructions were usually not necessary. However, there were occasions where verbal instructions were less effective than non-verbal instructions, and others where non-verbal instructions were less effective than verbal instructions.

Individual differences of course interact with age differences Caviola et al. studied children's strategy choices in solving complex subtraction problems, and investigated the effects of grade and of variations in problem complexity. Third-grade children (mean age 105.9 months) and fifth-grade children (mean age 129.8 months) solved multi-digit subtraction problems and described their solution strategies. In one experiment (*n* = 155; *n* = 76 in third grade; and *n* = 79 in fifth grade), they chose their strategies spontaneously, and in another experiment (*n* = 175; *n* = 88 in third grade; and *n* = 87 in fifth grade), they were asked to choose between specified strategies. Fifth-grade children tended to use more efficient strategies, such as retrieval and decomposition, while third-grade children were more likely to use less efficient strategies such as counting and to rely more on the written right-to-left solution algorithm. However, all strategies were used by children in both age groups, and strategy choice was influenced by problem characteristics including problem complexity and presentation format.

Deng et al. carried out one of the few studies in this Research Topic that focussed on individual differences in adults. They investigated the Spatial Numerical Association of Response Codes (SNARC) effect in 240 adults using a parity judgment task (odd vs. even?) and a magnitude classification task (greater or smaller than 5?) for the eight numbers from 1 to 9 except for 5, which were randomly presented one at a time. Each task was carried out over 16 phases, divided into two blocks with a short interval between them, in each of which all eight items were administered. The order of the blocks was counterbalanced across participants, Detailed analyses were carried out of the changes in response times and the SNARC effect across the range of numbers and over the time course across the 16 phases. The SNARC effect emerged earlier and stayed more stable in magnitude classification task than in the parity task during the time course. It also increased over the time course in the magnitude classification task, whereas it fluctuated up and down over the time course in the parity task.

(2) The componential nature of arithmetic: how different aspects of arithmetic may diverge from one another, and how they may be influenced by different factors.

Baten and Desoete examined individual differences in primary school children's mathematics learning by combining antecedent (A), opportunity (O), and propensity (P) indicators within the Opportunity-Propensity Model (Byrnes and Miller, 2016). They studied the mathematical abilities of 114 primary school children (in grades 3–6, age range 8–12) with (*n* = 61) and without (*n* = 53) mathematical learning disabilities in relation to questionnaires given to them and to their parents and teachers. Results indicated that children with and without mathematical difficulties showed significant differences in personality, motivation, temperament, subjective well-being, self-esteem and self-perceived competence, and that there were also significant differences in parental aspirations for them. As regards antecedent (A) factors, parental aspirations explained about half of the variance in fact retrieval speed in children without mathematical learning disabilities, and socio-economic status was a strong predictor of procedural accuracy in both groups. Teachers' experience (number of years that they had taught mathematics) was considered as an Opportunity (O) factor and explained about 6% of the variance in mathematical abilities. Propensity (P) indicators explained between 52 and 69% of the variance, with intelligence as the most significant predictor overall. Indirect effects suggested that the predictors were interrelated and highlighted the value of including A, O, and P indicators in a comprehensive model. Moreover, different A, O, and P indicators seemed to be important for fact retrieval speed compared to procedural accuracy, supporting componential theories of arithmetic.

Salminen et al. studied the early number skill profiles of 440 pre-primary Finnish children (with a mean age of 75 months), longitudinally over three points in an 8-month period. They modeled latent performance-level profile groups for three early number skill components that had been previously found to predict arithmetic (symbolic number comparison, mapping, and verbal counting skills). Four profile groups were found: lowest-performing (6%), low-performing (16%), near-average-performing (33%), and high- performing (45%). The groups differed significantly in all three number skill components and in basic arithmetic, with the lowest-performing children showing particular difficulties in the number comparison and mapping tasks, perhaps indicating problems with accessing the semantic meaning of symbolic numbers. The profiles appeared to be mostly stable over the 8-month period.

Ganor-Stern focussed in particular on the nature of exact calculation vs. computational estimation. She investigated 4th (*n* = 33), 5th (*n* = 33), and 6th grade pupils (*n* = 33) and college students (*n* = 25) performance on exact calculation and computational estimation tasks involving two-digit multiplication problems. The estimation tasks involved stating whether the result of each problem was larger or smaller than a given reference number. Older children were more accurate than younger children on the calculation task, but there were no age differences among the children for accuracy on the estimation task. There were no age differences among the children for reaction times on either task, but adults were faster than children on both. At all ages, within group variability in accuracy was greater for the exact calculation task than in the computation estimation task. Accuracy on the two tasks did not correlate strongly. The findings suggest exact calculation and computational estimation may at least in part involve different skills.

One important distinction between components of numeracy is that between symbolic and non-symbolic representations of number (Lyons et al., 2012; Schneider et al., 2017). Li et al. investigated the development of children's symbolic and non-symbolic representations of number. Participants were 253 four-to-eight-year-old children from the first and second grades of two primary schools. The researchers studied their symbolic and non-symbolic representations, their ability to map between the two types of representation, and their mathematical ability. Non-symbolic representation emerged earlier than symbolic representation, but by the age of 6, children performed equally well at both types. Children of 6 or older were able to map between symbolic and non-symbolic quantities. Path analyses showed a direct effect of children's symbolic numerical skills on mathematical performance, but non-symbolic numerical skills only affected mathematical performance indirectly via symbolic skills. The influences of symbolic and non-symbolic numerical skills on mathematical performance both decreased with age.

(3) The relationship between arithmetic and cognitive characteristics.

Wei et al. investigated the predictive role of three core executive functions (inhibition, shifting, and working memory) on the growth of mathematical skills. They carried out a 3-year longitudinal study with 179 Chinese children from second to fifth grade. In second grade with a mean age of 97.89 months, they were assessed on the above executive functions, as well as non-verbal IQ, speed of processing and number sense. Each year from second through fifth grade, they were tested on arithmetic accuracy and fluency. Structural equation modeling showed that non-verbal IQ, speed of processing, and number sense all predicted the intercept in arithmetic accuracy, while working memory was the only executive function to predict the rate of growth in arithmetic accuracy. Number sense, speed of processing, inhibition, and shifting were all significant predictors of the intercept in arithmetic fluency; but none of the executive functions predicted rate of growth in arithmetic fluency. Thus, the study suggests both that executive functions predict mathematical learning and performance, and that different executive functions may predict different aspects of mathematics.

Ding et al. studied the roles of working memory and two domain-specific factors—single-step mental addition skills, and strategy use—in multi-step mental addition in two groups of Chinese elementary students. In Study 1 (*n* = 40), they studied the effect on strategy types of task manipulations involving schema automaticity (whether intermediate sums added up to decades, e.g., convert 16 + 27 to 16 + 24 = 40 + 3 = 43) and working memory load (two steps vs. four steps). In Study 2 (*n* = 43), they studied the effect on strategy types of task manipulations involving schema automaticity (one-time vs. two-time regrouping) and working memory load (partial vs. complete decomposition). Results of both studies suggested that shorter response time on single-step mental addition, choice of easier strategies, and phonological working memory performance were all associated with shorter response time on multi-step mental addition. The findings in both studies highlighted the important role of the phonological loop in mental addition in Chinese children.

Siemann and Petermann discussed explanations for developmental dyscalculia, and in particular, the question of whether mathematical ability depends purely on domain-general cognitive abilities, or requires an innate number sense. They suggest that the controversy arises from ambiguity about what number sense is. They argue that it is common for early number competence to be used as a proxy for innate magnitude processing, even though it requires some knowledge of the number system (i.e., the sequence of symbols, counting words or Arabic numerals, to represent number). Thus, most studies that refer to “non-symbolic” number processing are in fact referring to tasks requiring some symbolic knowledge as well. The authors suggest that developmental dyscalculia is in fact due to a conglomerate of deficits rather than a single deficit.

Reeve et al. studied the extent to which the variability in the time children took to solve single digit addition (SDA) problems predicted their later ability to solve more complex mental addition problems; and whether children with deficits could thus be distinguished from those with typical or delayed mathematical acquisition. One hundred sixty-four children were tested on four occasions over a 6-year period starting from the age of five. They were tested on digit span, visuospatial working memory and non-verbal IQ; speed in naming single numbers and letters; speed in subitizing one to three dots; and on four occasions, speed and accuracy on a 12-item single digit addition test. At the end of the study, the children, by then aged 11, were given a double-digit mental addition test. The researchers conducted a latent profile analysis to determine if there were different variability patterns over time with regard to single digit addition. There were three distinct variability patterns. In a typical acquisition pathway, mean reaction times were relatively low and reaction time variability decreased over time. In a delayed pathway, both mean reaction time and reaction time variability started out as high, but decreased over time. In a deficit pathway, mean reaction time and reaction time variability remained high throughout the study. The deficit pathway differed significantly from the other pathways in subitizing, but not in domain-general cognitive abilities or in double-digit addition. The researchers concluded that it is important to study individual differences in reaction time variability longitudinally, and that the results highlight the importance of subitizing ability as a diagnostic index for mathematical difficulties.

Van Luit and Toll studied 84 Dutch pupils between the ages of 8 and 18, with a diagnosis of developmental dyscalculia. They looked at the prevalence in this group of deficits in four cognitive characteristics: planning skills, naming speed, short-term and/or working memory, and attention. They found that the commonest deficit was in naming speed (in particular, naming numbers), followed by deficits in short-term/working memory and planning skills. Deficits in attention were the least common.

Wang et al. investigated whether children with mathematical difficulties also experience deficits in executive functions, and whether these could be explained by lower-level deficits in processing speed. They assessed 84 children of approximately 10 years: 23 children with mathematical difficulties alone; 30 children with combined mathematical and reading difficulties; and 31 typically developing children. The children were given tests of reading, mathematics, inhibition, attentional shifting, working memory and processing speed. The children with mathematical difficulties performed worse than typically developing children on all executive function tasks. Children with only mathematical difficulties performed similarly to the children with combined mathematical and reading difficulties, except in attentional shifting, where the former performed better. However, group differences in executive functions disappeared after controlling for processing speed. Thus, it appears that most deficits in executive function, shown by Chinese children with mathematical difficulties can be accounted for by lower-level deficits in processing speed.

Mathematical ability is also considered to be influenced by language factors including both linguistic ability (Pimperton and Nation, 2010; Bjorn et al., 2016) and language background (Miura et al., 1993; Krinzinger et al., 2011; Klein et al., 2013; Dowker and Nuerk, 2016; Dowker and Li, 2019). In particular, speakers of languages with more transparent counting systems such as Chinese seem to find some aspects of mathematics easier than speakers of languages with less transparent counting systems such as English. McClung and Arya studied individual differences in 23,220 Chinese and English fourth-grade pupils mathematics achievement. They used a subset of the 2011 Progress in International Reading and Literacy Study (PIRLS) and Trends in International Mathematics and Science Study (TIMSS) data from students who were tested in Chinese or English in nine countries. Their overall scores for mathematics and reading were assessed; and their scores specifically on the Number content of the test were used to assess whether they did or did not have mathematical difficulties. Hierarchical linear modeling analyses suggested that the main effect of language on mathematical performance remained significant once their categorization as having vs. not having mathematical difficulties was added to the model. However, the effect of language on mathematical performance appeared to be especially salient in the presence of mathematical difficulties; suggesting that linguistic factors such as counting system transparency may be particularly important for children who are struggling with numeracy.

(4) The relationships between mathematical performance and mathematics anxiety.

Kucian et al. examined the relationship between negative emotion toward mathematics and arithmetical performance in children with and without developmental dyscalculia. They studied 172 primary school children (76 with developmental dyscalculia and 96 controls). They used an affective priming task, which consisted of a simple addition or subtraction true/false decision task preceded by a prime, which consisted of words with either positive, negative, neutral affect, and words related to mathematic. It was expected that performance children with developmental dyscalculia would be slower and less accurate if preceded by a mathematics prime. In fact, neither group showed a negative mathematics priming effect, though children with dyscalculia showed lower mathematics performance than controls, and also showed more mathematics anxiety in an explicit questionnaire. Explicit mathematics anxiety correlated negatively with performance in both groups. This suggests that in primary school children, mathematics anxiety and its relation to performance may be more reliably measured by an explicit questionnaire than by a priming task. This is also suggested for university students in an unpublished study by (Dowker and Parker, 2013).

Some of the studies have looked at how the relationship between mathematical performance and mathematics anxiety may be mediated by other cognitive factors. Zhang et al. studied mathematical word-problem solving and its relation to several cognitive and affective factors in 116 third-grade Chinese children with a mean age of 9.6 years. They found that after controlling for age and non-verbal intelligence, mathematical word problem solving correlated positively with working memory, reading comprehension and mathematical fact fluency, and negatively with mathematics anxiety. It also correlated negatively with reading anxiety, but this relationship turned out to be fully mediated by mathematics anxiety.

Soltanlou et al. studied the relationships between mathematics anxiety, visuospatial memory and mathematical learning. Twenty-five 5th graders with a mean age of 11.13 years underwent seven training sessions of multiplication over the course of 2 weeks. After the sessions, children were faster and more accurate in solving trained problems than untrained problems. Children who were both high in mathematics anxiety and low in visuospatial working memory showed worse learning than other children. This was shown specifically for accuracy, but not for reaction time. It is interesting that children with poor visuospatial working memory as well as high mathematics anxiety showed this effect. This may be because mathematics anxiety increases the load on working memory, but this only has a negative impact if working resources are already limited. We would also suggest that, as some studies have indicated (e.g., DeCaro et al., 2010), mathematics anxiety may exert its strongest effect on verbal working memory, so that visuospatial working memory may compensate for this in individuals with good visuospatial working memory, but not in those with poor visuospatial working memory.

Júlio-Costa et al. studied mathematics anxiety from a different perspective. They investigated aspects of the molecular-genetic contribution to mathematics anxiety. They looked in particular at the COMT Val158Met polymorphism, which affects dopamine levels in the prefrontal cortex, and has been found to be associated with anxiety (Hosák, 2007). Two copies of the valine allele (Val/Val) is associated with lower dopamine availability, and two copies of the methionine allele (Met/Met) with higher dopamine availability. The researchers assessed 389 school children aged 7–12 years for intelligence, numerical estimation, arithmetic achievement and mathematics anxiety and genotyped them for the COMT Val158Met polymorphism. No significant main effects were found on any of the genotype related measures. However, there were significant interactions between gender and genotype for IQ and mathematics anxiety. IQ scores were higher in Met/Met girls than in girls with at least one valine allele, though the genotype effects were not significant for boys. In the case of mathematics anxiety, heterozygous individuals tended to score close to the average, regardless of gender. Homozygous boys for either val/val or met/met showed significantly less mathematics anxiety than heterozygous boys and homozygous girls for either val/val or met/met showed significantly more mathematics anxiety than heterozygous girls.

(5) Applications of the study of individual differences in arithmetic to the development or improvement of educational practices for arithmetic teaching as a whole and/or interventions for children with difficulties.

Cerda et al. compared two teaching approaches to formal and informal mathematical reasoning with two groups of young Spanish schoolchildren (*n* = 229), aged four and five. The ABN method (Open Algorithm Based on Numbers; *n* = 147) was associated with better results than the CBC method (Closed Algorithms Based on Ciphers; *n* = 82), which is the usual approach in Spanish schools. Moreover, the effect was greater in children who received more instruction on skills considered as domain-specific predictors of later arithmetic, such as magnitude comparison and knowledge of cardinality.

Auer et al. pointed out that children have often been found to make suboptimal choices between mental and written strategies to solve division problems. In particular, lower-attaining pupils often use mental strategies where the use of written algorithms would be more efficient. They divided 147 sixth-grade pupils with low mathematics attainment into two training groups: one with explicit training to promote writing down calculations, and one which devoted a similar amount of time to practice, but without explicit targeting of strategy use. Both groups improved considerably from pretest to post-test with regard both to general performance and to selection of written strategies. However, the two training groups did not differ from one another.

Koponen et al. carried out an intervention study with elementary school children in grades 2 to 5 with poor calculation fluency (mean age: 114 months). The aim was to investigate the effects of strategy training focusing on derived fact strategies integrating factual, conceptual, and procedural arithmetic knowledge. Thus, 69 Finnish children were selected on the basis of scoring below the 20th percentile on a standardized mathematics test, and using counting-based strategies in an individual assessment. The children participated in a group based strategy training twice a week for 45 min over a 12-week period. In addition, they underwent two short weekly practice sessions for basic addition skills. Their addition fluency was assessed before and immediately after intervention, and at a 5-month post-intervention follow-up, and their progress was compared with that of two control groups: one that received a reading intervention and a business-as-usual group. The mathematics intervention group improved significantly more in addition during the intervention than either of the control groups. There was an increase in fact retrieval and derived fact strategies and a decrease in counting-based strategies in the mathematics intervention group, compared to the control groups. The effects did not, however, transfer to subtraction fluency. At 5-month follow-up the mathematics intervention group maintained their gains, but did not show further progress. They were still performing better on addition fluency than the reading intervention group, but were similar to the business-as-usual group.

Friso-van den Bos et al. divided 90 kindergarten children in the Netherlands, with a mean age of 5 years 8 months, into three groups: one trained on counting, one on number line placement, and one a business-as-usual control group. They were pre-tested and post-tested on arithmetic, counting, number lines, and number comparisons. The group trained on counting improved significantly more in arithmetic, counting, and number lines than the business-as-usual group. The group trained on number line use did not differ significantly on any measure from the business-as-usual group.

Björn et al. investigated Response to Intervention (RTI) methods in the USA and in Finland. The authors discuss the frameworks in the two countries from the point of view of assessment and instruction. They suggest that the Finnish framework is an example of support in mathematics learning that incorporates principles of RTI, such as systematized assessment and instruction, cyclic support, and modifiable instruction. Similarly, close monitoring of student progress is also at the core of RTI in the US. Informed decision making at all levels within the system (administrative, teacher, and parental; see Fuchs and Fuchs, 2005) is provided. The basic idea of RTI in the U.S. is that the school provides the child with research-based instruction while the child is in the general education environment, and the school adjusts the intensity or nature of assessment and instruction according to the student's progress (Fuchs and Fuchs, 2005). One important difference between the American and Finnish frameworks is that the American version was primarily developed for learning difficulty identification and the Finnish version was primarily intended to re-structure the existing support services for pupils struggling with mathematics. After analyzing the similarities and differences between the American and Finnish systems, the authors conclude by discussing possibilities for further refinements of the RTI approach in both countries.

## Conclusion

The studies in this volume support previous studies in indicating that there are marked individual differences in arithmetic at all ages from preschool to adulthood; that these appear to be related to domain-specific factors, domain-general factors and emotional factors, though there is still much controversy about how these factors interact. The studies also demonstrate that arithmetical cognition is composed of multiple components, though there may be controversy about how these are related to one another and which components are most important; and that these findings can be put to good use in developing interventions and methods of instruction. The studies also show that findings from different countries (e.g., the UK, USA, China, and Finland) often converge to give similar results and conclusions.

Further research should expand the age groups studied, to include more work with toddlers at one end and adults at the other, and to incorporate more longitudinal studies. There should also be more work on how different components of arithmetical thinking interact with, and predict, one another and how this may change with age and instruction. There should also be further work on how domain-specific and domain-general factors interact with each other at a given time and longitudinally and the extent to which both numerical abilities and so-called domain-general abilities may be influenced by context. On the other hand, one might wonder whether the terms “domain-specific” and “domain-general” are ideal as they may sometimes be misleading. For example, it is not always what constitutes as a “domain”; so-called domain-specific predictors of one ability, such as phonological awareness being predictive for reading, are also predictive of performance in another domain, i.e., arithmetic (e.g., De Smedt et al., 2010); measures of executive function always involve the processing of certain types of stimuli (e.g., numbers), and these more specific processing differences in itself may underlie individual differences. Cultural influences on both mathematical performance and mathematics anxiety should also be explored. Finally, further progress needs to be made in the development and evaluation of interventions, and in systematically investigating whether different types of intervention may be differentially effective for children with different mathematical profiles.

## Author Contributions

All authors listed have made a substantial, direct and intellectual contribution to the work, and approved it for publication.

### Conflict of Interest

The authors declare that the research was conducted in the absence of any commercial or financial relationships that could be construed as a potential conflict of interest.
